# Editorial: Big data in systems biology

**DOI:** 10.3389/fsysb.2026.1905528

**Published:** 2026-07-14

**Authors:** Holger Husi

**Affiliations:** Nottingham Ningbo China Beacons of Excellence Research and Innovation Institute, University of Nottingham Ningbo China (UNNC), Ningbo, China

**Keywords:** data integration, disease analysis, integrative analysis, meta-analysis, multi-modal data, multi-omcis, systems biology

## Introduction

Systems biology, which is defined by its aim to integrate molecular layers into coherent models, is now being reshaped by the scale, diversity, and complexity of modern biological data. The six articles featured in this collection illustrate this multi-disciplinary field which is undergoing rapid transformation and is expanding both in depth and breadth, ranging from multimodal single-cell technologies to multilayer network models, from epidemiological meta-analyses to statistical innovations for correlated genomic data, and from doublet detection to immune-cell annotation. Across these contributions, a unifying theme emerges, which is that big data is no longer a challenge to be managed, but rather it is the substrate from which biological meaning can be extracted. However, the path forward requires methodological innovation, conceptual integration, and a commitment to interpretability.

## Statistical approaches: learning Gaussian graphical models from correlated data

Song et al. address a methodological gap in systems biology, which is the difficulty of inferring network structure from correlated observations. Gaussian Graphical Models (GGMs) are widely used to infer conditional dependencies, but traditional implementations assume independent observations, which are rarely met in longitudinal or clustered studies. Their proposed cluster-based bootstrap algorithm offers a statistically rigorous solution, controlling type I error without sacrificing power. Using polygenic risk scores from the Long Life Family Study, they demonstrate that ignoring within-cluster correlation can lead to inflated false positives, whereas their novel approach yields more reliable network structures. This contribution is a reminder that big data is only as useful as the statistical frameworks that support its analysis, and as datasets grow in complexity, methodological innovation becomes not optional but essential.

## Cellular systems: single-cell technologies for multimodal omics measurements

Bai and Zhu provide a concise review of the transforming landscape of multimodal single-cell technologies. As they note, single-cell transcriptomics has revolutionised the ability to interrogate cellular heterogeneity, but it risks capturing only a “partial picture of the cells’ complex molecular networks”. Their review highlights the emergence of multimodal platforms capable of measuring combinations of RNA, chromatin accessibility, protein abundance, and spatial context within the same cell. This shift is not just technical, but also fundamentally conceptual. Multimodal data allow researchers to reconstruct regulatory hierarchies, infer causal relationships, and understand how molecular layers interact to produce distinct phenotypes. However, these technologies also generate unprecedented data volumes, which require computational frameworks capable of integrating signals across modalities without sacrificing interpretability. The challenge therefore is not only to measure more but also to understand more.

## Quality control: detecting doublets in single-cell data

Zhao et al. tackle a practical but critical issue in single-cell RNA-seq analysis, namely, doublet detection. Doublets, or instances where 2 cells are captured under a single barcode, can distort clustering, mislead annotation, and generate spurious biological conclusions. Their model-driven algorithm, scMODD, performs comparably to existing data-driven methods but with greater interpretability. Notably, they find that incorporating zero inflation does not improve performance, suggesting that “consideration of zero inflation is not necessary” for this task. This insight contributes to a growing recognition that not all complexities of scRNA-seq data require equally complex models.

## Cell type annotation algorithms: comparisons applied to datasets of immune response of COVID-19

Xu et al. compare 5 cell type annotation algorithms and apply them to PBMC datasets from COVID-19 patients. Using Azimuth, they identify significant depletion of plasmacytoid dendritic cells and strong activation of type I interferon signalling, which are consistent with known immunopathology of COVID-19. Their conclusion is that cell-based annotation methods outperform cluster-based approaches. This study highlights the importance of accurate annotation for downstream biological interpretation and reinforces the need for benchmarking as datasets and algorithms proliferate.

## Data integration: multilayer networks and digital twins

Chenel et al. demonstrate that multilayer network models offer a principled solution to the integration problem. Rather than concatenating omics layers or analysing them in isolation, multilayer networks preserve the structure, redundancy, and cross-talk inherent to biological systems. As the authors write, this framework “captures the modularity, redundancy and cross-talk between layers” and provides a scaffold for constructing personalized digital twins. Digital twins, computational replicas of individual biological systems, represent one of the most ambitious frontiers in systems biology. Their promise lies in simulating disease trajectories, predicting treatment responses, and enabling precision medicine at scale. But digital twins require models that are both mechanistic and interpretable. Multilayer networks, with their explicit representation of interactions across molecular strata, offer a compelling foundation.

## Epidemiology: population-scale inference

Cheuyem et al. extend the conversation from molecular systems to population-level dynamics through a systematic review and meta-analysis of mpox coinfections in Africa. Their findings reveal substantial prevalence of VZV–mpox (8.73%) and HIV–mpox (4.29%) coinfections, with significant heterogeneity across settings. Importantly, they note that coinfections are “far more prevalent in hospital-based environments” and that clinical manifestations vary by viral clade, with clades I and Ia associated with more severe systemic symptoms. This work underscores the importance of integrating clinical, epidemiological, and genomic data, an approach entirely aligned with systems biology principles. The authors’ call for strengthened diagnostic capacity and routine screening echoes a broader theme that data integration is not only a scientific challenge but a public health imperative.

## Conclusion

**FIGURE 1:**
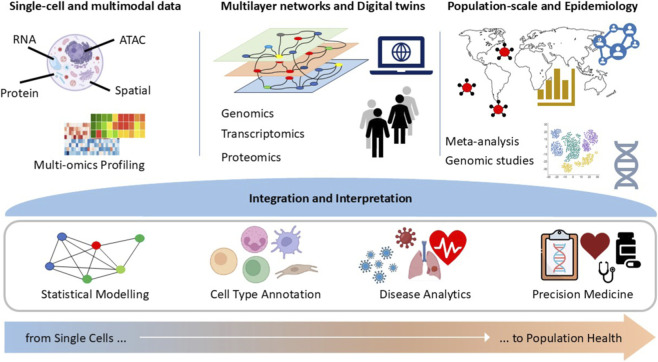
Systems biology and big data synthesis.

As summarised in Figure 1, the future of systems biology will depend on the ability to integrate the various layers of multifaceted data into a coherent whole, but as data become more complex, it is crucial that interpretability remains the core principle. As models become more powerful, they must reflect biological reality, and as applications expand, they must continue to be aligned with societal needs, particularly those related to public health readiness to precision medicine. The articles in this collection demonstrate that the field is rising to these challenges with creativity, rigour, and ambition, and the next decade promises even deeper integration of data, models, and applications, bringing us closer to a truly holistic understanding of biological systems.

